# TangShenWeiNing Formula Prevents Diabetic Nephropathy by Protecting Podocytes Through the SIRT1/HIF-1α Pathway

**DOI:** 10.3389/fendo.2022.888611

**Published:** 2022-05-26

**Authors:** Jing Chang, Jinsu Zheng, Xia Gao, Hengbei Dong, Haitian Yu, Mengxiu Huang, Zhencheng Sun, Xiaomeng Feng

**Affiliations:** ^1^ Department of Internal Medicine, Beijing Chao-Yang Hospital, Capital Medical University, Beijing, China; ^2^ Department of Traditional Chinese Medicine, Beijing Chao-Yang Hospital, Capital Medical University, Beijing, China; ^3^ Department of Endocrinology, Beijing Chao-Yang Hospital, Capital Medical University, Beijing, China; ^4^ Department of Reproductive Medicine, Beijing Obstetrics and Gynecology Hospital, Capital Medical University, Beijing, China; ^5^ Education Division, Beijing Chao-Yang Hospital, Capital Medical University, Beijing, China; ^6^ Department of Hepatobiliary, Beijing Chao-Yang Hospital, Capital Medical University, Beijing, China; ^7^ Department of Osteology, Beijing Chao-Yang Hospital, Capital Medical University, Beijing, China

**Keywords:** TangShenWeiNing formula, diabetic nephropathy, podocytes (MeSH: D050199), SIRT1, HIF-1α

## Abstract

**Background:**

Diabetic nephropathy (DN) represents a major complication of diabetes, and podocyte injury has a critical function in DN development. TangShenWeiNing formula (TSWN) has been demonstrated to efficiently decrease proteinuria and protect podocytes in DN. This work aimed to explore the mechanism by which TSWN alleviates DN and protects podocytes.

**Methods:**

The major bioactive components of TSWN were detected by mass spectrometry (MS) and pharmacological databases. Eight-week-old male C57BLKS/J db/m and db/db mice were provided pure water, valsartan, low dose TSWN, middle dose TSWN and high dose TSWN by gavage for 12 weeks, respectively.

**Results:**

MS and network pharmacology analyses suggested that TSWN might prevent DN through the sirtuin (SIRT)1/hypoxia-inducible factor (HIF)-1α pathway. Diabetic mice showed elevated urinary albumin in comparison with non-diabetic mice, and TSWN decreased urinary albumin in diabetic mice. Histological injury increased in the kidney in diabetic mice, which could be improved by TSWN. Fibrosis and collagen I expression were induced in the diabetic mouse kidney in comparison with the non-diabetic mouse kidney; TSWN alleviated these effects. Apoptosis and cleaved caspase-3 were induced in the diabetic mouse kidney in comparison with the non-diabetic mouse kidney, and TSWN blunted these effects. Podocytes were damaged in the diabetic mouse kidney, which was improved by TSWN. Podocin and nephrin amounts were decreased in the diabetic mouse kidney in comparison with the non-diabetic mouse kidney, and podocalyxin was increased in urine of diabetic animals in comparison with non-diabetic counterparts. After TSWN treatment, podocin and nephrin were raised in the diabetic mouse kidney, and urinary podocalyxin was depressed in diabetic animals. Diabetic mice had lower SIRT1 and higher HIF-1α amounts in kidney specimens in comparison with non-diabetic mice, and TSWN promoted SIRT1 and inhibited HIF-1α in the diabetic mouse kidney. Moreover, co-staining of SIRT1 and podocin revealed that SIRT1 decreased in podocytes from diabetic mice in comparison with those from non-diabetic mice, and TSWN elevated SIRT1 in podocytes.

**Conclusions:**

This study indicated that TSWN alleviates DN by improving podocyte injury through the SIRT1/HIF-1α pathway in diabetic mouse kidneys.

## Introduction

Diabetic nephropathy (DN) or diabetic kidney disease (DKD) represents a common complication of diabetes mellitus and a major cause of end-stage renal disease (1). Podocyte injury is the major pathogenesis of DN. Podocytes are highly specialized, terminally differentiated cells. Hyperglycemia leads to abnormalities of podocyte-associated proteins and signaling pathways, and podocyte apoptosis accelerates disease progression (2). It is known that podocytes have a limited renewal ability. Podocyte injury has been identified as a major event resulting in proteinuric kidney diseases and renal failure (3). Therefore, a treatment that reduces podocyte injury can reduce urinary albumin, delay kidney function damage, and prevent or ameliorate DN progression.

So far, there is no ideal preventive and treatment methods to effectively delay diabetic kidney damage and prevent podocyte injury. Conventional therapeutic strategies, including glycemic control, weight control, and blockage of the renin-angiotensin-aldosterone system, may not achieve satisfactory therapeutic effects in many clinical practices. On the other hand, attention is being paid to traditional Chinese medicine, which can be used as the first or alternative therapy for the treatment of DN with good clinical efficacy. Increasing attention is paid to the identification and molecular mechanisms of bioactive compounds of traditional Chinese medicine on diabetic renal protection (4).

TangShenWeiNing formula (TSWN) represents a traditional Chinese herbal formula developed by experts of the Department of Traditional Chinese Medicine, Beijing Chao-yang Hospital, Capital Medical University. TSWN has been utilized clinically for treating DN for more than two decades, with favorable effects. Recently, TSWN was shown to reduce proteinuria and protect podocytes in patients with DN (data unpublished), suggesting that TSWN administration may result in pronounced therapeutic effects on DN. However, the mechanism by which TSWN alleviates DN remains undefined.

Network pharmacological analyses have demonstrated that hypoxia-inducible factor (HIF)-1 constitutes one of the main targets of TSWN. HIF represents a heterodimer comprising a constitutively expressed β-subunit and at least one of the oxygen-dependent α-subunits, i.e., HIF-1α and -2α. HIF activity is mostly modulated by oxygen-associated proteolysis of α-subunits (5). It was recently demonstrated that HIF-1α plays a dual role in DN. Studies have shown that HIF-1α elicits a protective effect in physiological or pathological hypoxia or ischemia, such as DN. However, the mainstream belief among scientists is that elevated HIF-1α is involved in the pathological process and proteinuria of glomerular diseases in DN. Podocyte damage may be susceptible to the accumulation of HIF-1α (6). In a previous work, increased HIF by knockout of prolyl hydroxylase domain protein-2 (PHD2), a factor degrading HIF, enhances renal fibrosis (7). HIF-1α upregulation is involved in kidney injury, and its inhibition results in DN prevention in diabetic mice (5, 8).

Recent studies have documented that HIF-1α is regulated by many factors, and sirtuin (SIRT) 1 is a major regulating factor of HIF-1α (9). The SIRT family consists of SIRT1-SIRT7 in mammals, and shares the same 275-amino acid catalytic core region. SIRT1, located in the nucleus, is a nicotinamide adenine dinucleotide dependent deacetylase (10). SIRT1 is closely related to aging-related diseases, diabetes, vascular diseases and kidney diseases, and widely involved in the regulation of various intracellular processes, including apoptosis, metabolism and autophagy (10, 11). Meanwhile, SIRT1 has been identified as a novel molecular target for the prevention and treatment of kidney diseases. Previous studies have revealed that SIRT deficiency sensitizes Ang-II-induced renal fibrosis (12). SIRT1 could delay the progression of various kidney diseases by inhibiting apoptosis and fibrosis (13, 14). It was indicated that overexpression of SIRT1 in podocytes attenuates proteinuria and kidney injury in an animal model of diabetes (15). On the contrary, the decrease of SIRT1 in podocytes was shown to increase urinary protein and exacerbate renal injury (16).

SIRT1 binds to and deacetylates HIF-1α at Lys674, which inactivates HIF-1α and suppresses HIF-1α targets (17). It was verified that upregulation of SIRT1 inhibits the development of diabetic microvascular diseases *via* downregulation of HIF-1α (18). Moreover, DN prevention could be achieved by regulating the SIRT1/HIF-1α pathway (19).

Based on network pharmacology analyses and previous studies, TSWN might regulate the SIRT1/HIF-1α pathway to prevent DN. However, whether TSWN alleviates DN by regulating the SIRT1/HIF-1α pathway in podocytes remains unclear. The current work aimed to assess how TSWN prevents DN.

## Materials and Methods

Assays involving animals had approval from the Animal Ethics Committee of Beijing Chao-Yang Hospital, Capital Medical University, and followed the animal care guidelines of Beijing Chao-Yang Hospital, Capital Medical University.

### Medicines and Reagents

TSWN (Patent No. 202111331292.3 under review by the State Intellectual Property Office of China) contains 13 Chinese herbs, including Huangqi (ASTRAGALI RADIX) 20g (20/185), Taizishen (PSEUDOSTELLARIAE RADIX) 15g (15/185), Danggui (ANGELICAE SINENSIS RADIX) 10g (10/185), Dihuang (REHMANNIAE RADIX) 20g (20/185), Shanzhuyu (CORNI FRUCTUS) 10g (10/185), Shanyao (DIOSCOREAE RHIZOMA) 15g (15/185), Tianhuafen (TRICHOSANTHIS RADIX) 15g (15/185), Gouqizi (LYCII FRUCTUS) 15g (15/185), Danshen (SALVIAE MILTIORRHIZAE RADIX ET RHIZOMA) 15g (15/185), Fuling (PORIA) 15g (15/185), Zexie (ALISMATIS RHIZOMA) 10g (10/185), Taoren (PERSICAESE MEN) 15g (15/185), and Gancao (GLYCYRRHIZAE RADIX ET RHIZOMA) 10g (10/185). TSWN was purchased from Pharmacy of Chao-Yang Hospital, Capital Medical University.

### Ultra-High Performance Liquid Chromatography-Tandem Mass Spectrometry Analysis

TSWN extraction and storage were carried out as follows. TSWN underwent centrifugation (3000 rpm, 5 min). The resulting supernatant underwent filtration through a 0.45 μm PTFE membrane and storage at 4°C until assessment.

A UPLC-MS comprising a HESI-II probe was utilized for mass spectrometry analysis. The operating parameters were as follows: positive and negative HESI spray voltages, 3.7 and 3.5 kV, respectively; oven temperature, 300°C; sheath and auxiliary gas, nitrogen; collision gas, nitrogen; pressure, 1.5 mTorr; flow rate, 0.3 mL/min; column temperature, 45°C. Data collection and processing utilized Masslynx 4.1 and the Scientific Information System. The main components were quantitated with the UPLC system equipped with an Acquity UPLC column (2.1 mm × 100 mm, 1.8 µm).

### Detection of Pharmaceutical Components

In this study, to determine the effective targets of TSWN, the traditional Chinese medicine systems pharmacology (TCMSP) database (https://www.tcmsp-e.com/) (20) was utilized for assessing all herbs in TSWN, and the components with oral bioavailability (OB) ≥ 0.3 and drug-likeness (DL) ≥ 0.18 (21, 22) were selected. Then, the intersection of the selected components by network pharmacology and mass spectrometry results was considered.

In order to retrieve the disease targets, the online mendelian inheritance in man (OMIM) database (https://omim.org/) (23), drug bank database (https://go.drugbank.com/) (24), discover genes internet (DisGeNET) data (25) (https://www.disgenet.org/) and Gene Cards database (https://www.genecards.org/) (26) were used, and “Diabetic Nephropathy” was used as a search term in these databases.

Venn diagrams were performed for TSWN’s active component targets as well as disease targets. The intersection of drug and disease targets was selected, and Kyoto Encyclopedia of Genes and Genomes (KEGG) enrichment analysis was carried out in the Metascape database (https://metascape.org/) (27). All figures were generated with the R 4.1.1 statistical software.

### Animal Experiments

Male C57BLKS/J db/m and db/db mice (7 weeks old) were provided by Nanjing Biomedical Research Institute of Nanjing University, Nanjing, China. The animals were assigned to six groups, including the db/m, db/db, db/db+V, db/db+TSWN-L, db/db+TSWN-M and db/db+TSWN-H groups (n = 6 per group).

Totally 185 g crude drugs of TSWN underwent soaking in 400 ml pure water for decoction to yield a concentration of 2 g/mL. TSWN in the present research was utilized at low, medium and high doses of 6.01, 12.02 and 24.05g/kg, respectively, twice per day in the db/db+TSWN-L, db/db+TSWN-M and db/db+TSWN-H groups, respectively (the medium dose was based on the dosage commonly administered to adult humans). The db/db+V group was administered 10.29 mg/kg of valsartan (Beijing Novartis Pharmaceutical, Beijing, China; dose based on the dosage commonly administered to adult humans) dissolved in pure water once per day and pure water alone once per day. The db/m and db/db groups were administered pure water alone twice per day. The volumes of intragastric administration of different groups were consistent with db/db+TSWN-H group by supplementing pure water. All the above gavage treatments were carried out for 12 weeks from 8 weeks of age.

The conditions were as follows: n = 3/cage; light cycle, 12-h light/dark cycle (lights on 08:00 - 20:00 h); temperature, 22 ± 1°C; humidity, 40%; freely available water and food; litter replacement, once a day.

Following a 12-week administration, the animals were housed in individual metabolic cages for taking urine samples, and anesthesia was performed by intraperitoneally injecting Rompun 10 mg/kg (Bayer Korea, Ansan, Gyeonggi-Do, Korea) and Zoletil 30 mg/kg (Virbac, Carros, France) in combination at week 20. Blood samples were collected from the left ventricle and kept at -80°C for subsequent analysis. Euthanasia was followed by kidney removal.

### Blood and Urine Tests

Blood and urine parameters were measured as follows. Blood glucose (GLU) level was detected with a HemoCue B-Glucose kit (HemoCue AB, Angelholm, Sweden). Insulin (INS) levels were detected with a radioimmunoassay kit (Linco Research, St Charles, MO, USA). Total cholesterol (TC) and triglycerides (TG) levels were detected by an auto-analyzer (Wako, Osaka, Japan). Blood urea nitrogen (BUN) was measured with a iStat-Kit (HESKA, Fort Collins, MO, USA). Serum and urine creatinine concentrations were detected by HPLC (Beckman Instruments, Fullerton, CA, USA). Urine albumin concentration was detected by an immunoassay (Bayer, Elkhart, IN, USA). Urine albumin-to-creatinine ratio (UACR) was derived as urine albumin/urine creatinine (μg/mg). Serum creatinine and alanine aminotransferase (ALT) amounts were examined with an automatic biochemical analyzer (Olympus AU480, Japan). Urinary podocalyxin levels in mice were measured by enzyme linked immunosorbent assay (ELISA) (Exocell, Philadelphia, PA, USA). The above assays followed the directions of the respective manufacturers.

### Light Microscopy

Kidney tissue specimens underwent fixation with 10% formalin (SF93-20; Fisher Scientific, Pittsburgh, PA, USA). Histological features were assessed by hematoxylin and eosin (H&E; Servicebio, Wuhan, China) and Periodic Acid Schiff (PAS; Servicebio) staining. Fibrosis was assessed by the ratio of fibrotic area to total area detected by Masson’s trichrome (Servicebio) and Sirius red (Servicebio) staining. Kidney apoptosis was examined by TUNEL (Servicebio). Kidney specimens underwent embedding in frozen optimal cutting temperature compound (Fisher HealthCare, Houston, TX, USA) and sectioning at 8 µm for immunostaining. The specimens underwent incubation with primary antibodies targeting SIRT1 (1:100; Abcam, Cambridge, MA, USA) and podocin (1:100; Sigma, Shanghai, China). This was followed by incubation with second antibodies conjugated with fluorescein isothiocyanate (1:500). For quantitation, 10 random high-power fields in mouse kidney samples were assessed with Image J (NIH, Bethesda, MD, USA).

### Transmission Electron Microscopy

Three kidney specimens per group were sliced through the hilum in a longitudinal fashion. Then, kidney specimens underwent mincing into rectangular pieces of approximately 1 mm, fixation with 2.5% glutaraldehyde (4 h at 4°C) and four rinsing steps with 0.1 mol/L phosphate buffer saline (PBS; 15 min each). After fixation with 1% citrate (2 h) and two rinsing steps with 0.1 mol/L PBS (5 min each), dehydration was carried out with acetone at 50, 70, 90 and 100%, successively (15 min each). Specimens underwent infiltration with acetone and plant fats at ratios of 1:1 and 2:1 for 2 h, respectively, followed by overnight infiltration with pure resin. After Epon 812 resin embedding, ultrathin sections at 60-70 nm were obtained with an Ultracut R microtome. Uranium acetate and lead nitrate were utilized for staining before observation.

### Western Blot

Samples were randomly selected from the six groups. Assays were performed thrice. Kidney cortex tissue specimens underwent homogenization, followed by a 10-minute centrifugation (16000×g at 4°C). A bicinchoninic acid protein assay kit (Pierce Co, Rockford, IL, USA) was utilized for protein quantitation. Totally 20 µg of protein per sample underwent separation by 10% sodium dodecyl sulfate polyacrylamide gel electrophoresis gel and transfer onto polyvinylidene difluoride (PVDF) membranes. After blocking (5% skimmed milk in Tris-buffered saline), overnight incubation was carried out with primary antibodies targeting collagen I (1:1000; Abcam, Cambridge, MA, USA), cleaved caspase-3 (1:1000; Abcam), podocin (1:1000; Abcam), nephrin (1:1000; Abcam), HIF-1α (1:1000; Novus Bio, Littleton, CO, USA), SIRT1 (1:1000; Abcam) and β-actin (1:1000; Cell Signaling, Danvers, MA, USA). Next, a 2-hour incubation with secondary antibodies linked to horseradish peroxidase (1:5000; Santa Cruz, CA, USA) was carried out. Quantitation was performed by densitometry with the image acquisition and analysis software (Bio-Rad).

### Ribonucleic Acid Extraction and Quantitative Reverse Transcriptase Polymerase Chain Reaction

RNA extraction from kidney tissue specimens utilized TRIzol (Invitrogen, Carlsbad, CA, USA) using standard protocols as directed by the manufacturer. qRT-PCR was carried out with QuantiTect SYBR Green PCR Kit (Qiagen, Valencia, CA). Primers were: SIRT1, 5’-GCTGACGACTTCGACGACG-3’ (sense) and 5’-TCGGTCAACAGGAGGTTGTCT-3’ (antisense); HIF-1α, 5’-CTCGGCGAAGCAAAGAGT-3’ (sense) and 5’-GCCATCTAGGGCTTTCAG-3’ (antisense); β-actin, 5’-CATCCGTAAAGACCTCTATGCCAAC-3’ (sense) and 5’-ATGGAGCCACCGATCCACA-3’ (antisense).

### Statistical Analyses

Data are mean ± SEM. Multiple groups were compared by one-way ANOVA. SPSS 22.0 (SPSS, Inc., Chicago, IL, USA) was utilized for data analysis, and two-sided *P*<0.05 was deemed statistically significant.

## Results

### Potential Targets of TSWN Effects on DN

**Figure 1:**
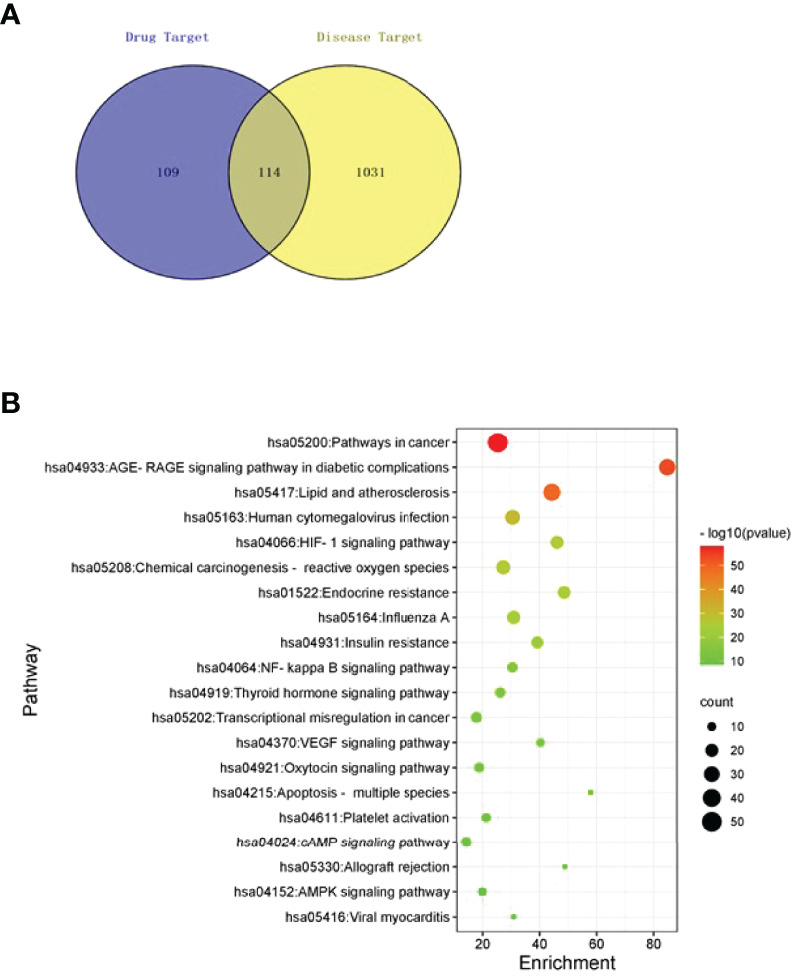
Network pharmacological analyses. **(A)** Venn diagram of targets of TSWN formula and targets of diabetic nephropathy. **(B)** Pathway enrichment analysis of differential genes by TSWN formula treatment on diabetic nephropathy. The vertical axis represents the names of the 20 selected pathways, the color of the dot represents the -log10(P) value, the size represents the number of genes, and the horizontal axis is the enrichment of pathways.

Totally 15 bioactive components (**Table 1**) and 223 effective targets of TSWN as well as 1145 targets of DN were screened out by mass spectrometry (**Supplementary 1**) and network pharmacology analysis (**Supplementary 2A** **–** **D**). Venn diagrams were generated for TSWN’s effective targets and DN targets (**Figure 1A**). The intersection of TSWN and DN targets was selected, and KEGG enrichment analysis was performed by using the Metascape database (https://metascape.org/) (27). The results are shown in **Figure 1B**, and HIF-1 was one of the main targets of TSWN. Since HIF-1α is regulated by SIRT1 (16, 17), systems pharmacology revealed that TSWN might prevent DN by regulating the SIRT1/HIF-1α pathway.

**Table 1:** Active components of TSWN formula.

Mol ID	Name	OB (%)	DL
MOL000422	Kaempferol	44.88	0.24
MOL000098	Quercetin	46.43	0.28
MOL001689	Acacetin	34.97	0.24
MOL000006	Luteolin	36.16	0.25
MOL008400	Glycitein	50.48	0.24
MOL001942	Isoimperatorin	45.46	0.23
MOL002776	Baicalin	40.12	0.75
MOL007088	Cryptotanshinone	52.34	0.4
MOL007154	Tanshinone IIa	49.89	0.4
MOL000291	Poricoic acid B	30.52	0.75
MOL000289	Pachymic acid	30.62	0.75
MOL002565	Medicarpin	49.22	0.34
MOL004328	Naringenin	59.29	0.21
MOL004908	Glabridin	53.25	0.47
MOL004949	Isolicoflavonol	45.17	0.84

### Mouse Biophysical Features

**Figure 2:**
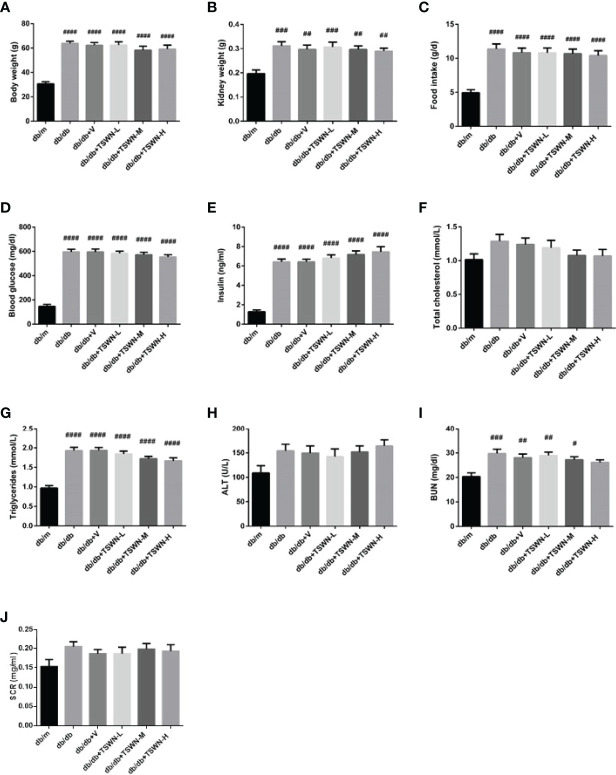
Physical and biochemical characteristics of mice. **(A)** Body weight. **(B)** Kidney weight. **(C)** Food intake. **(D)** Fasting blood glucose level. **(E)** Fasting Insulin level. **(F)** Fasting total cholesterol. **(G)** Fasting triglycerides. **(H)** Alanine aminotransferase (ALT). **(I)** Blood urea nitrogen (BUN). **(J)** Serum creatinine (SCr). n = 6 mice/group. ^#^P < 0.05, ^##^P < 0.01, ^###^P < 0.001, ^####^P < 0.0001 vs db/m group. Db/m, db/m mice; db/db, db/db mice; db/db+V, db/db mice with valsartan treatment; db/db+TSWN-L, db/db mice with low dose TSWN treatment; db/db+TSWN-M, db/db mice with middle dose TSWN treatment; db/db+TSWN-H, db/db mice with high dose TSWN treatment. Data are means ± S.E.M.

In this study, body and kidney weights, food intakes, blood glucose amounts, insulin levels and triglycerides were significantly higher in the db/db, db/db+V, db/db+TSWN-L, db/db+TSWN-M and db/db+TSWN-H groups compared with the db/m group. These parameters were comparable in the db/db, db/db+V, db/db+TSWN-L, db/db+TSWN-M and db/db+TSWN-H groups (**Figures 2A–E, G**). It was found that blood urea nitrogen (BUN) level was significantly higher in the db/db, db/db+V, db/db+TSWN-L and db/db+TSWN-M groups compared with the db/m group, and there was no significant difference in BUN among db/db, db/db+V, db/db+TSWN-L, db/db+TSWN-M and db/db+TSWN-H groups (**Figures 2I**). All groups had comparable total cholesterol, ALT and serum creatinine (SCR) levels (**Figures 2F, H, J**).

### Renal Phenotype of Mice

**Figure 3:**
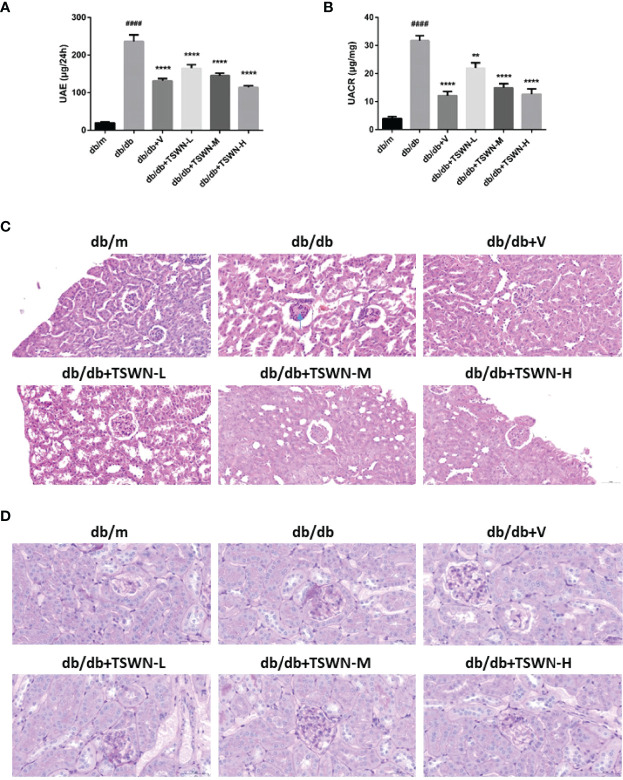
Renal phenotype of mice. **(A)** Urinary albumin excretion (UAE). **(B)** Urinary albumin-to-creatinine ratio (UACR).** (C)** Representative photographs of mouse kidneys by Hematoxylin & Eosin (H&E) staining. **(D)** Representative photographs of mouse kidneys by Periodic Acid Schiff (PAS) staining. n = 6 mice/group. ^####^P < 0.0001 vs db/m group; **P < 0.01 vs db/db group. Db/m, db/m mice; db/db, db/db mice; db/db+V, db/db mice with valsartan treatment; db/db+TSWN-L, db/db mice with low dose TSWN treatment; db/db+TSWN-M, db/db mice with middle dose TSWN treatment; db/db+TSWN-H, db/db mice with high dose TSWN treatment. Data are means ± S.E.M.

As depicted in **Figure 3**, the db/db group had elevated urinary albumin excretion (UAE) and UACR in comparison with the db/m group. In addition, the db/db+V, db/db+TSWN-L, db/db+TSWN-M and db/db+TSWN-H groups had markedly reduced UAE and UACR values in comparison with the db/db group (**Figures 3A, B**). H&E staining showed mesangial basement membrane thickening and KW nodule formation in the db/db group. PAS staining showed that the mesangial glomerular basement membrane was remarkably increased in db/db group. However, valsartan or TSWN treatment prevented renal pathological changes in mice with experimental diabetes (**Figures 3C, D**).

### Renal Fibrosis in Mice

**Figure 4:**
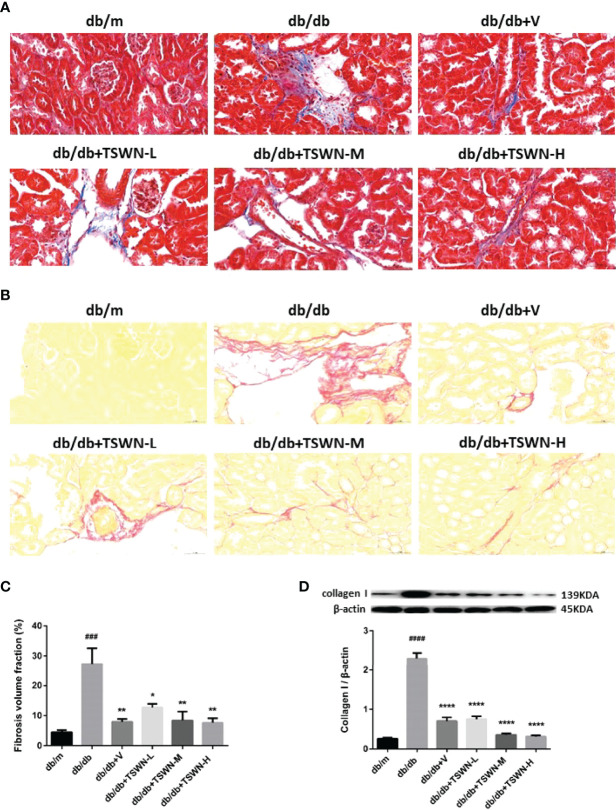
Fibrosis in mouse kidneys. **(A)** Representative photographs of renal fibrosis measured by Masson’s staining. **(B)** Representative photographs of renal fibrosis measured by Sirius red staining. **(C)** Quantification of renal fibrosis. **(D)** Representative photographs and quantification of collagen I in mouse kidneys detected by western blot. n = 6 mice/group. ^###^P < 0.001, ^####^P < 0.0001 vs db/mgroup; *P < 0.05, **P < 0.01, ****P < 0.0001 vs db/db group. Db/m, db/m mice; db/db, db/db mice; db/db+V, db/db mice with valsartan treatment; db/db+TSWN-L, db/db mice with low dose TSWN treatment; db/db +TSWN-M, db/db mice with middle dose TSWN treatment; db/db+TSWN-H, db/db mice with high dose TSWN treatment. Data are means ± S.E.M.

Renal fibrosis was assessed by Masson’s staining and Sirius red staining (**Figures 4A–C**). Both assays demonstrated that diabetes markedly induced renal fibrosis in mice, which was suppressed by valsartan or TSWN. Immunoblot further revealed that diabetes upregulated fibrosis associated protein-collagen I in the mouse kidney, which was alleviated by valsartan or TSWN (**Figure 4D**).

### Renal Apoptosis in Mice

**Figure 5:**
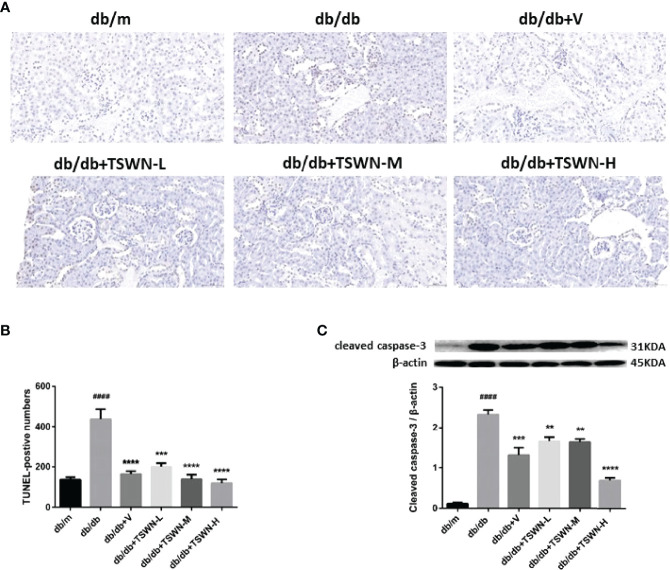
Apoptosis in mouse kidneys. **(A, B)** Representative photographs and quantification of renal apoptosis measured by TUNEL assay. **(C)** Representative photographs and quantification of cleaved caspase-3 in mouse kidneys detected by western blot. n = 6 mice/group. ^####^P < 0.0001 vs db/m group; **P < 0.01, ***P < 0.001, ****P < 0.0001 vs db/db group. Db/m, db/m mice; db/db, db/db mice; db/db+V, db/db mice with valsartan treatment; db/db+TSWN-L, db/db mice with low dose TSWN treatment; db/db+TSWN-M, db/db mice with middle dose TSWN treatment; db/db+TSWN-H, db/db mice with high dose TSWN treatment. Data are means ± S.E.M.

Subsequently, we measured apoptosis in the mouse kidney by TUNEL, and cleaved caspase-3 by Western blot. The TUNEL assay demonstrated that diabetes markedly induced mouse renal apoptosis, which was prevented by valsartan or TSWN (**Figures 5A, B**). Meanwhile, immunoblot demonstrated that diabetes upregulated apoptosis-associated cleaved caspase-3 in the mouse kidney, while valsartan or TSWN treatment suppressed renal cleaved caspase-3 expression in diabetic animals (**Figure 5C**).

### Podocyte Injury in Mouse Kidneys

**Figure 6:**
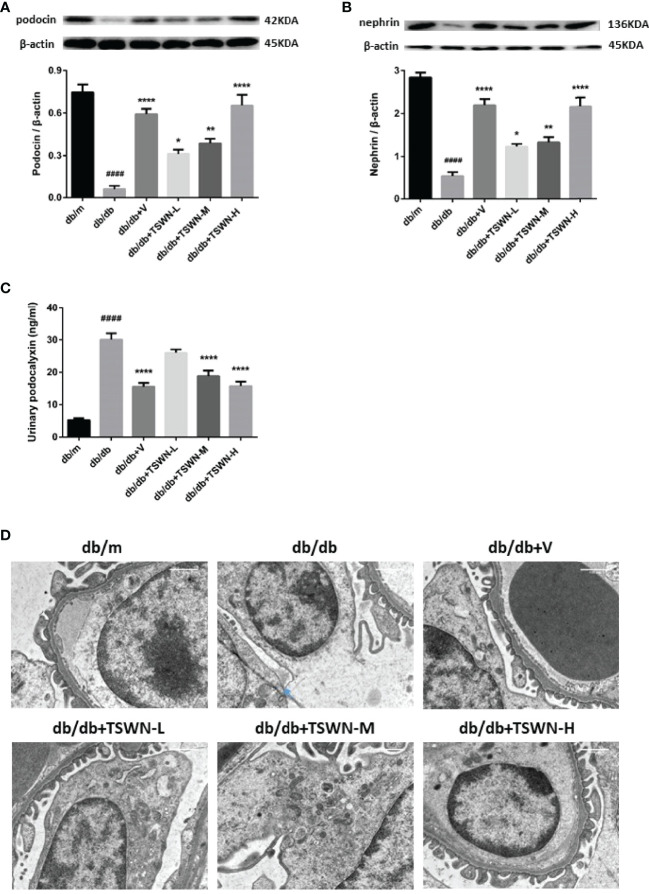
Podocytes in mouse kidneys. **(A)** Representative photographs and quantification of podocin in mouse kidneys measured by western blot. **(B)** Representative photographs and quantification of nephrin in mouse kidneys measured by western blot. **(C)** Quantification of urinary podocalyxin measured by enzyme linked immunosorbent assay (ELISA). **(D)** Representative photographs of podocytes in mouse kidneys detected by transmission electron microscopy. n = 6 mice/group. ^####^P < 0.0001 vs db/m group; *P < 0.05, **P < 0.01, ****P < 0.0001 vs db/db group. Db/m, db/m mice; db/db, db/db mice; db/db+V, db/db mice with valsartan treatment; db/db+TSWN-L, db/db mice with low dose TSWN treatment; db/db+TSWN-M, db/db mice with middle dose TSWN treatment; db/db+TSWN-H, db/db mice with high dose TSWN treatment. Data are means ± S.E.M.

Podocyte injury has an important function in DN. Therefore, we investigated TSWN’s effects on podocytes in mouse kidneys. As depicted in **Figure 6**, the protein levels of the podocyte markers podocin and nephrin were lower in the kidneys of the db/db group in comparison with the db/m group (immunoblot), and podocalyxin, another marker of podocytes, had elevated urine amounts in the db/db group compared with the db/m group, as detected by ELISA. Meanwhile, podocin and nephrin were raised in the diabetic mouse kidney, and urine podocalyxin was decreased in mice with experimental diabetes after valsartan or TSWN treatment (**Figures 6A–C**). Next, podocyte morphology was examined by transmission electron microscopy. Damaged podocytes with perforation were found in the kidneys of db/db mice, while podocyte injury was improved in the kidneys of diabetic mice after valsartan or TSWN treatment (**Figures 6D**).

### Assessment of SIRT1/HIF-1α Pathway in Mouse Kidneys

**Figure 7:**
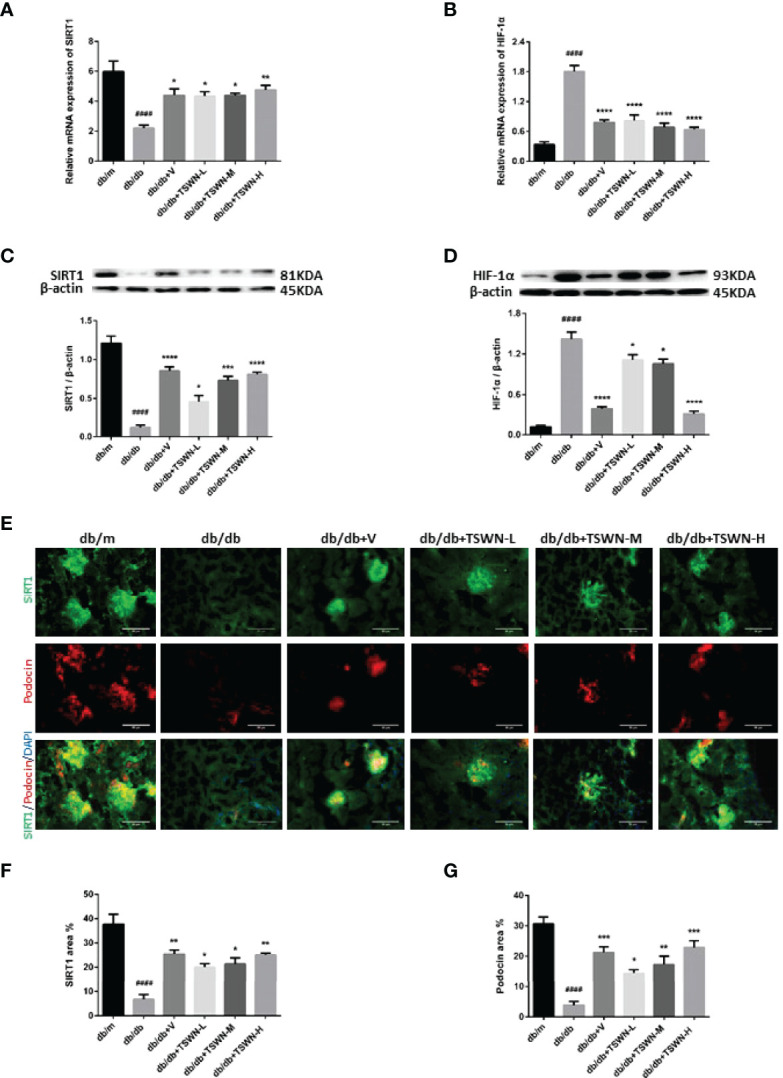
SIRT1 and HIF-1a in mouse kidneys. **(A)** Messenger ribonucleic acid (mRNA) expression of SIRT1 in mouse kidneys by quantitative reverse transcriptase polymerase chain reaction (RT-PCR). **(B)** MRNA expression of HIF-1a in mouse kidneys by RT-PCR. **(C)** Representative photographs and quantification of SIRT1 in mouse kidneys measured by western blot. **(D)** Representative photographs and quantification of HIF-1a in mouse kidneys measured by western blot. **(E–G)** Representative photographs and quantification of co-staining of SIRT1 and podocin in mouse kidneys. n = 6 mice/group. ^####^P < 0.0001 vs db/m group; *P < 0.05, **P < 0.01, ***P < 0.001, ****P < 0.0001 vs db/db group. Db/m, db/m mice; db/db, db/db mice; db/db+V, db/db mice with valsartan treatment; db/db+TSWN-L, db/db mice with low dose TSWN treatment; db/db+TSWN-M, db/db mice with middle dose TSWN treatment; db/db+TSWN-H, db/db mice with high dose TSWN treatment. Data are means ± S.E.M.

Then, renal SIRT1 and HIF-1α amounts were assessed in mice. As shown in **Figure 7**, the db/db group showed lower SIRT1 and elevated HIF-1α in kidneys measured by both qRT-PCR and immunoblot compared with the db/m group. After valsartan or TSWN treatment, SIRT1 was upregulated and HIF-1α was suppressed in diabetic mouse kidneys (**Figures 7A–D**). Moreover, the immunostaining fractions of renal SIRT1 and podocin were decreased in the db/db group in comparison with the db/m group, and valsartan or TSWN treatment raised SIRT1 and podocin in diabetic mouse kidneys. Furthermore, co-staining of SIRT1 and podocin showed that SIRT1 amounts were decreased in podocytes of the db/db group versus db/m animals, whereas valsartan or TSWN treatment increased SIRT1 levels in the podocytes of diabetic mouse kidneys (**Figures 7E–G**).

## Discussion

This work demonstrated that UAE and UACR were increased in diabetic mice, and diabetes accelerated pathological changes, promoted fibrosis and apoptosis, and induced podocyte injury in mouse kidneys. Furthermore, SIRT1 was decreased and HIF-1α was increased in diabetic mouse kidneys compared with non-diabetic mouse kidneys. TSWN reduced UAE and UACR, improved renal pathological changes, inhibited renal fibrosis, decreased renal apoptosis and prevented podocyte injury in diabetic mice. Importantly, TSWN regulated the SIRT1/HIF-1α pathway in the podocytes of diabetic mouse kidneys.

DN represents a commonly detected microvascular complication of diabetes, accounting for adverse clinical outcome (28). Treatment options for DN mainly reduce albuminuria to improve the prognosis of clinical adverse events (29). At present, drugs commonly used to prevent DN and reduce albuminuria in clinic, including angiotensin receptor blockers (ARB), reduce renal fibrosis and apoptosis, and protect podocytes (30, 31). In addition, ARB can delay DN through the SIRT1/HIF-1α pathway (32, 33). TSWN, a traditional Chinese herbal formula, contains 13 Chinese herbs. TSWN provides an effective outcome of therapeutic effects in patients with DN by preventing podocyte injury. Based on mass spectrometry and network pharmacology, TSWN might prevent DN and decrease urinary albumin *via* the HIF-1 pathway.

The pathological characteristics of DN include a variety of structural and functional changes in the kidney, leading to albuminuria (34). In the present study, hyperglycemia damaged the kidneys of diabetic mice, leading to increased albuminuria and aggravated kidney histology in mice with experimental diabetes. However, TSWN treatment could reduce albuminuria and improve kidney histology in animals with experimental diabetes, suggesting TSWN’s curative effect on DN.

Fibrosis represents a hallmark of progressive chronic renal disorders, potentially leading to renal failure. High blood glucose upregulates the expression fibrotic factors, further resulting in damaged glomerular filtration barrier and causing DN (29). In the current study, diabetes caused fibrosis and increased collagen I amounts in the mouse kidney, and these effects were alleviated by TSWN, suggesting that TSWN reduces renal fibrosis in diabetic mice.

Hyperglycemia induces apoptosis in podocytes (35). Accumulating evidence demonstrates that apoptosis accelerates the pathogenesis of DN (36). In the present study, diabetes enhanced apoptosis and upregulated cleaved caspase-3 in the mouse kidney. However, TSWN treatment was confirmed to prevent renal apoptosis, downregulating cleaved caspase-3 in the diabetic mouse kidney. These findings confirmed that TSWN could reduce renal apoptosis in diabetic mice.

Podocytes are cells with high level of differentiation, found outside the glomerular basement membrane. They form the last line of defense for the glomerular filtration barrier (37). Since podocytes show limited capabilities of repair and regeneration, the degree of podocyte injury is considered the main prognostic determinant of DN (29). Podocytes are critical for renal function, and constitute the primary focus in multiple renal disorders, especially DN. Damage of podocytes contributes to the accumulation of podocyte-derived cell debris and podocyte-specific molecular targets in urine, which could be detected by specific tests (38). In this study, diabetic mice had lower levels of podocin and nephrin in renal tissues and higher levels of podocalyxin in urine compared with non-diabetic mice. After TSWN treatment, the levels of podocin and nephrin in renal tissues were increased and the levels of podocalyxin in urine were decreased in diabetic mice. In addition, podocyte damage was observed by transmission electron microscopy in diabetic mouse kidneys, which was improved by TSWN treatment. These findings indicated TSWN reduces podocyte injury in diabetic animals.

According to mass spectrometry and network pharmacology, TSWN might prevent DN and decrease urinary albumin *via* the HIF-1α pathway. HIF-1α has a known association with DN, and could play a protective role in DN (6). Accumulating evidence reveals that elevated HIF-1α leads to DN and podocyte injury. Under hyperglycemic conditions, elevated expression of HIF-1α, a transcriptional factor mediating hypoxia adaptation, could stimulate renal fibrosis and proteinuria (39, 40). HIF-1α expression is accompanied by renal fibrosis in diabetes, and HIF-1α upregulation can cause renal injury (41). Conversely, experimental knockout or inhibition of HIF-1α attenuates renal fibrosis (42, 43). Recent reports have documented that HIF-1α is regulated by SIRT1, a nicotinamide adenine dinucleotide dependent deacetylase that deacetylates HIF-1α at Lys674 and inactivates HIF-1α, leading to the suppression of HIF-1α target genes (17). SIRT1 was recently identified as a novel molecular target for the prevention and treatment of several renal diseases, including DN. Overexpression of SIRT1 in podocytes attenuates proteinuria and kidney injury in an animal model of diabetes (15). Furthermore, SIRT1 regulates renal apoptosis and fibrosis. Hyperglycemic conditions caused SIRT1 in the kidney to decline, inducing apoptosis and fibrosis (10, 11, 13–15). Then, all these effects promoted the development of DN. In the present study, diabetic mice had lower SIRT1 and higher HIF-1α in the kidney in comparison with non-diabetic mice, and TSWN promoted SIRT1 and inhibited HIF-1α in diabetic mouse kidneys. Moreover, co-staining of SIRT1 and podocin revealed that SIRT1 was decreased in the podocytes of diabetic mouse kidneys in comparison with non-diabetic mouse kidneys, and SIRT1 was elevated in the podocytes of diabetic mouse kidneys upon TSWN treatment. These results suggested that TSWN prevents DN by modulating the SIRT1/HIF-1α pathway in the podocytes of diabetic mouse kidneys.

## Conclusions

In summary, this study indicated that TSWN plays an important role in DN treatment, and reduces albuminuria through the regulation of SIRT1/HIF-1α signaling in the podocytes of diabetic mouse kidneys. Further study of the therapeutic mechanism of TSWN in DN should be explored in the future.

## Data Availability Statement

The original contributions presented in the study are included in the article/**Supplementary Material**. Further inquiries can be directed to the corresponding author.

## Ethics Statement

The animal study was reviewed and approved by Animal Ethics Committee of Beijing Chao-Yang Hospital, Capital Medical University.

## Author Contributions

JC: Design, Experimentation, Statistics, Article revision. JZ: Statistics, Article revision. XG: Experimentation. HD: Experimentation. HY: Experimentation. MH: Experimentation. ZS: Experimentation. XF: Design, Experimentation, Statistics, Article revision. All authors contributed to the article and approved the submitted version.

## Funding

This work was supported by grants from Chinese National Natural Science Foundation (No. 81700713) and from Capital’s Funds for Health Improvement and Research, Beijing, PR China (No. CFH 2022-3-20311) to XF, and from the Scientific Research and Cultivation Project of Beijing Municipal Hospital (No. PX2022009) to JC.

## Conflict of Interest

The authors declare that the research was conducted in the absence of any commercial or financial relationships that could be construed as a potential conflict of interest.

## Publisher’s Note

All claims expressed in this article are solely those of the authors and do not necessarily represent those of their affiliated organizations, or those of the publisher, the editors and the reviewers. Any product that may be evaluated in this article, or claim that may be made by its manufacturer, is not guaranteed or endorsed by the publisher.
